# Supplementary material for: Genomic Epidemiology of Salmonid Alphavirus in Norwegian Aquaculture Reveals Recent Subtype-2 Transmission Dynamics and Novel Subtype-3 Lineages
Source: Viruses. 2021 Dec 20;13(12):2549. doi: 10.3390/v13122549 (PMC8705410; doi:10.3390/v13122549)
Supplement: Supplementary file 1 [file viruses-13-02549-s001.zip › Supplementary Information.pdf]

Supplementary Information

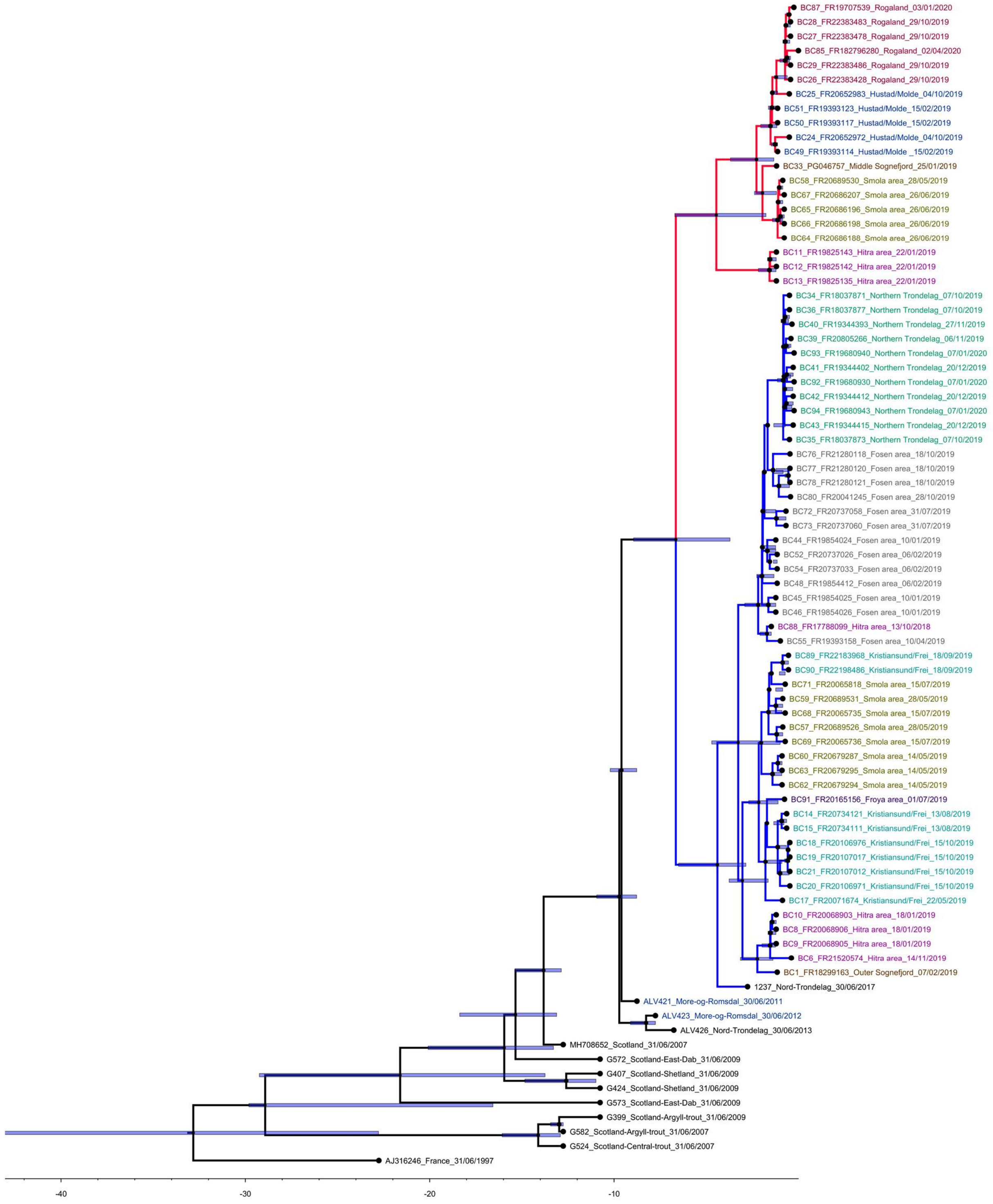

Figure S1. Bayesian time-calibrated phylogenetic tree used in Figure 4, providing sample names and accompanying sample dates, along with the 95% highest posterior density interval for divergence times at every node (light blue bar). The SAV2a and SAV2b clades are highlighted with red and blue branches, respectively.

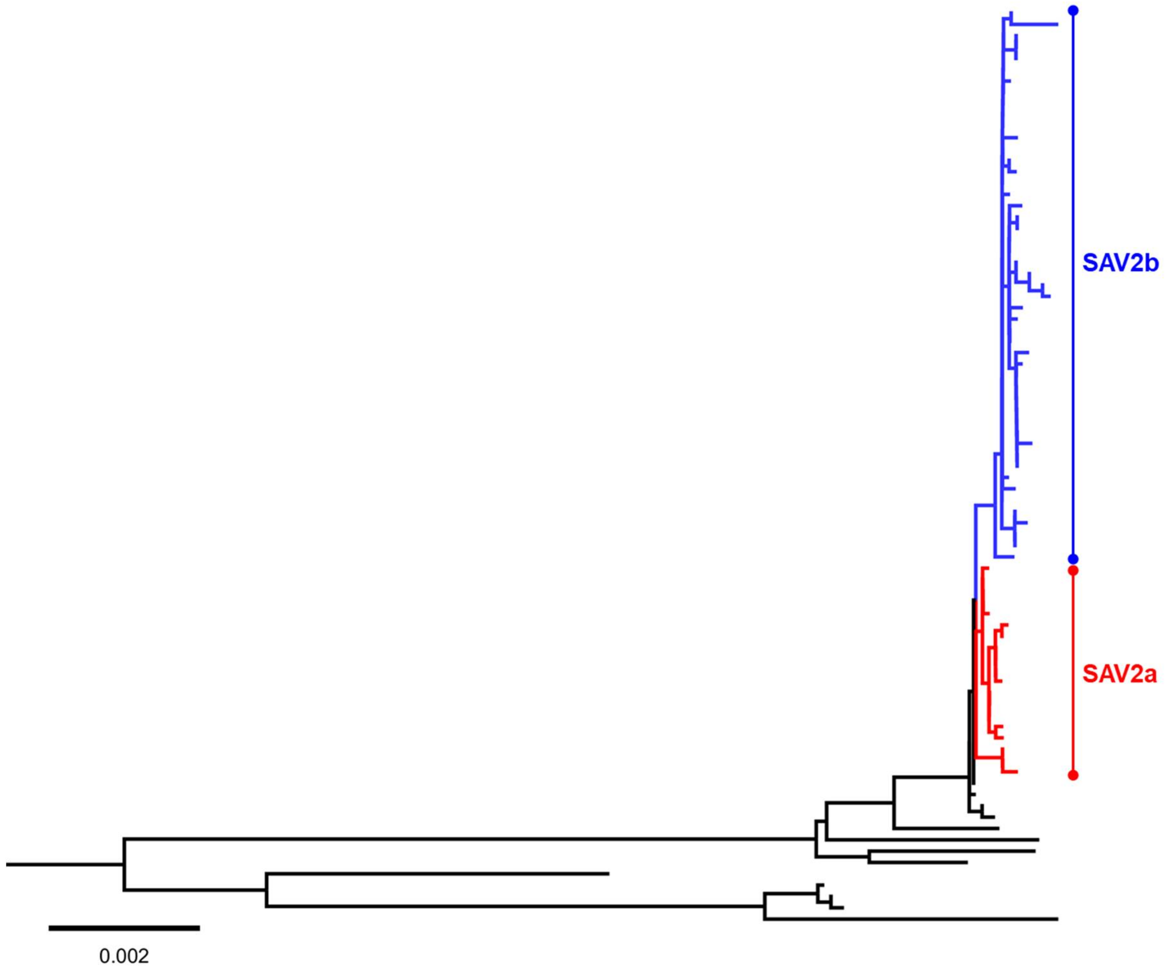

Figure S2. Mid-point rooted maximum likelihood phylogenetic tree summarizing the evolution of SAV2 in Norwegian aquaculture. The data represents 81 SAV2 sequences, inclusive of 68 generated by Nanopore sequencing in this study with average length of 10,600bp (Table S1), and 14 additional SAV2 genome sequences (Table S2). The tree was generated in IQ-TREE using the best-fitting nucleotide substitution model (TIM2+F+I). The scale bar shows the number of modelled substitutions per site.

Supplementary Note S1: scripts used in the study:

```
#Scripts used to generate consensus genome sequences from cleaned, barcoded fastq files
#########################################
#map 96 barcoded fastq files against the reference containing 8 #genome sequences and generate indexed bamfiles
###################################


INPUT=$(ls ./*.fastq.gz | awk "NR == $SGE_TASK_ID")
PREFIX="$(basename $INPUT)"
GENOME= 'sav_genomes.fasta'


minimap2 -ax map-ont -t 2 ${GENOME} $PREFIX | samtools sort -o $PREFIX.sorted.bam -T $PREFIX.tmp


samtools index $PREFIX.sorted.bam


#####################################################
#get coverage across genome for all the 96 barcodes
#################################################


for i in {01..96};
do
	while read -r a b c;
	do
		samtools coverage -r "$a":$b-$c ./BC"$i"_trimmed_final.fastq.gz.sorted.bam;
	done <genome_details >barcode"$i"_coverage.txt;
done


#####################################
#get coverage per amplicon across each SAV2 annd SAV3 genome for #96 barcodes
##############################


for i in {01..96}; # sav2 genome
```

```
do
	while read -r a b;
	do
		samtools coverage -r "SAV2_MH708652":$a-$b
./BC"$i"_trimmed_final.fastq.gz.sorted.bam;
	done <sav2_amplicon_details >barcode"$i"_sav2_coverage.txt;
done


for i in {01..96};#sav3 genome
do
	while read -r a b;
	do
		samtools coverage -r "SAV3_AY604238":$a-$b
./BC"$i"_trimmed_final.fastq.gz.sorted.bam;
	done <sav3_amplicon_details >barcode"$i"_sav3_coverage.txt;
done


#################
### generate consensus sequences for SAV2 and SAV3 genomes
#################

INPUT=$(ls ./*.sorted.bam | awk "NR == $SGE_TASK_ID")
PREFI="$(basename $INPUT)"
PREFIX="${PREFI%_trimmed_final.fastq.gz.sorted.bam}"
SAV2= 'sav2.fasta'
SAV3= 'sav3.fasta'

samtools  mpileup -uf ${SAV2} $PREFI | \
bcftools call -c --ploidy 1 | vcfutils.pl vcf2fq -d 50 >"$PREFIX"_SAV3_consensus.fastq
#rerun it for SAV3 for SAV3 consensus
```
